# Transcriptional control of anthocyanin biosynthetic genes in extreme phenotypes for berry pigmentation of naturally occurring grapevines

**DOI:** 10.1186/1471-2229-7-46

**Published:** 2007-08-30

**Authors:** Simone D Castellarin, Gabriele Di Gaspero

**Affiliations:** 1 Istituto di Genomica Applicata, Parco Scientifico e Tecnologico Luigi Danieli, via Jacopo Linussio 51, 33100 Udine, Italy; 2 Dipartimento di Scienze Agrarie e Ambientali, University of Udine, via delle Scienze 208, 33100 Udine, Italy

## Abstract

**Background:**

Fruit coloration of red-skinned grapevines is mainly due to anthocyanin pigments. We analysed a panel of nine cultivars that included extreme phenotypes for berry colour, ranging from green (absence of anthocyanins) to red, purple, violet and blue. Expression of six genes of the anthocyanin pathway coding for flavanone-hydroxylase (*F3H*), flavonoid 3'-hydroxylase (*F3'H*), flavonoid 3',5'-hydroxylase (*F3'5'H*), UDP-glucose:flavonoid-3-*O*-glucosyltransferase (*UFGT*), glutathione-*S*-transferase (*GST*), *O*-methyltransferase (*OMT*) and four transcription factors (*MybA*, *MybB*, *MybC*, *MybD*) was analysed by quantitative RT-PCR at four developmental stages from before the onset of ripening until full maturity and compared to anthocyanin metabolites.

**Results:**

Total anthocyanin content at full maturity correlated well with the cumulative expression of *F3H*, *UFGT *and *GST *throughout ripening. Transcripts of the last two genes were absent in the green-skinned cultivar 'Sauvignonasse', also known as 'Tocai friulano', and were at least 10-fold less abundant in pale red cultivars, such as 'Pinot gris' and 'Gewürztraminer', compared to fully coloured cultivars. Predominance of tri-hydroxylated anthocyanins (delphinidin, petunidin and malvidin) in cultivars bearing dark berries with violet and blue hue was associated with higher ratios of *F3'5'H/F3'H *transcription, compared to red-skinned cultivars. Higher levels of *OMT *transcripts were observed in berries of cultivars that accumulated methoxylated forms of anthocyanins more abundantly than non-methoxylated forms.

**Conclusion:**

Colour variation of the grape berry conforms to a peculiar pattern of genotype-specific expression of the whole set of anthocyanin genes in a direct transcript-metabolite-phenotype relationship. Cumulative mRNA levels of the structural genes and their relative abundance throughout ripening explained *per se *the final phenotype for anthocyanin content, anthocyanin composition, colour intensity and colour hue of grapes at berry maturity.

## Background

Regulation of anthocyanin biosynthesis has been studied across a number of flowering plants thanks to the availability of colour mutants that have facilitated the dissection of complex regulatory networks. Many studies have concerned themselves with the genetic control of colour phenotypes in floral organs of ornamentals (mainly *Antirrhinum majus *and *Petunia *× *hybrida*) or in kernels of *Zea mays *and seed teguments of *Arabidopsis thaliana*, which has led to a comprehensive view of the common features and the species-specific peculiarity of anthocyanin regulation in those plants [1-6]. In fruit trees, the genetics underlying the presence or absence of anthocyanin pigmentation has been elucidated in bilberry [7], grapevine [8,9] and apple [10]. However, studies pertaining to the quantitative and qualitative variation of anthocyanins in fruit and the resulting shifts in colour have only scratched the surface of the regulatory network by separately analysing the role of single genes [11-13] or single transcription factors [14,15].

Grapevine is a fruit crop that encompasses a wide phenotypic variation in berry colour. Plant adaptation to different environments and centuries of human selection have produced numerous genotypes in which the intensity and the hue of red coloration vary extensively. A mixture of variations in anthocyanin content and in the relative proportion of different anthocyanins can produce extreme phenotypes for skin pigmentation. Anthocyanin concentration in epidermal cells correlates well with the darkness of berry colour. According to [16], anthocyanin concentration ranged from 6.2 to 26 mg kg^-1 ^of berry in a panel of 64 pigmented cultivars. Anthocyanin profile and hue might vary dramatically as well. We have previously demonstrated that the ratio of blue tri-hydroxylated to red di-hydrohylated anthocyanins is under transcriptional control of flavonoid 3'(5')-hydroxylase genes (*F3'H *and *F3'5'H*) and it correlates well with the evolution of colour hue throughout ripening in the cultivar 'Merlot' [11]. All red cultivars investigated so far synthesise all five grapevine anthocyanins (cyanidin, peonidin, delphinidin, petunidin and malvidin) [16,17]. This means that all cultivars express functional *F3'H *and *F3'5'H *genes for the synthesis of 3'4'-OH and 3'4'5'-OH anthocyanins as well as *O*-methyltransferases (*OMT*) for the methylation of primary anthocyanins. These genes, alongside other key genes of the core pathway, are assumed to be differentially regulated in different genetic backgrounds. The biological questions we aimed to address are: does most regulation of the anthocyanin biosynthesis in ripening fruit occur at transcriptional level in a similar way as it occurs in other species in pigmented organs other than fruit ? Are the distinct patterns of expression of structural genes sufficient to explain the observed variation of contrasting colour phenotypes peculiar to each cultivar ?

Red-to-blue colour variation across grapevine cultivars has evolutionary, technological and health implications of fascinating interest. It is generally assumed that red coloration was the most primitive form of reproductive organs in plants that synthesise anthocyanins. Anthocyanins appeared approximately 120 million years ago [18,19] and naturally served for the recruitment of seed dispersers and in UV protection. Unlike roses and carnations, which still do not naturally display blue colouration, most species including grapevine have later acquired the capability of synthesising anthocyainic blue pigments. According to this view, the gene *F3'H *coding for the enzyme that leads to red anthocyanins is ancestral to the gene *F3'5'H *coding for the enzyme that converts red into blue anthocyanins [20]. Based on phylogenetic analysis, most *F3'5'H *arose from *F3'H *by gene duplication before the divergence of angiosperms and gymnosperms but in the documented case of the Asteraceae family this event occurred repeatedly and much more recently in the evolutionary time scale [20]. This raises the questions of how and when genes for blue coloration appeared in the grape genome. *F3'H *and *F3'5'H *currently known in the grape genome split in two different branches of a phylogenetic tree, that included also *F3'H *and *F3'5'H *from other plants [11]. *F3'5'H *from the grape genome grouped together with the homologous *F3'5'H *from other species rather than with the paralogous *F3'H *from grape. This feature is predictive of an ancient origin of the *F3'5'H *found in the present-day grapevines which was already present in the palaeo-ancestor before the split of major dicot lineages. More recently, *F3'5'H *has undergone further evolution in the grape genome, as witnessed by the structural complexity of the genomic region on the linkage group 6 containing *F3'5'H *[11].

Anthocyanin composition has a technological impact on the colour of must obtained from a given cultivar. Colour of pure anthocyanins shifts progressively from red to blue as the number of substituted groups on the B-ring increases and as methoxyl groups replace hydroxyl groups [21]. After must fermentation, anthocyanin stability in wines is threatened by a number of factors (light exposure, fluctuations in storage temperature, oxygen, enzymatic activities, *etc*.) which might lead to a premature deterioration of colour. The number and the pattern of the hydroxyl and methoxyl groups on the B-ring also affect the reactivity of the moiety. Cyanidin, delphinidin and petunidin have orto-di-phenolic groups which enhance susceptibility to oxidation [22]. Methoxylated anthocyanins, such as peonidin and malvidin, are more stable. The relative number of hydroxyl and methoxyl groups also affects polarity and solubility of the corresponding anthocyanin in aqueous and hydro-alchoolic solutions such as must and wine, respectively.

The whole class of polyphenol compounds, including anthocyanins, present in red grapes and wines is regarded as a powerful source of ROS scavengers [23,24]. However, the benefit for human health of each compound depends on its bioavailability and its antioxidant capacity. Dietary anthocyanins are adsorbed as intact 3-monoglucosides by a bilitranslocase in the epithelial cells of the gastric mucosa. For instance, the affinity of this carrier to each one of the five 3-monoglucoside anthocyanins varies within a 6-fold range [25]. This greatly affects the biological value of food and beverages depending not only on their total anthocyanin content but also on their peculiar anthocyanin profile.

In this paper, we report on transcript-metabolite-phenotype relationships between anthocyanin genes, anthocyanins and berry colour across cultivars of *Vitis vinifera*. The regulation of six genes of the anthocyanin biosynthetic pathway (*F3H*, *F3'H*, *F3'5'H*, *UFGT*, *OMT*, *GST*) as well as four related transcription factors (*MybA*, *MybB*, *MybC*, *MybD*) were monitored at four stages of ripening. The correlation between gene transcriptional levels, anthocyanin content/profile and skin pigmentation was assayed in nine cultivars encompassing most of the extreme variation for berry colour known in naturally occurring grapevines.

## Results

### Anthocyanin content

**Figure 1:**
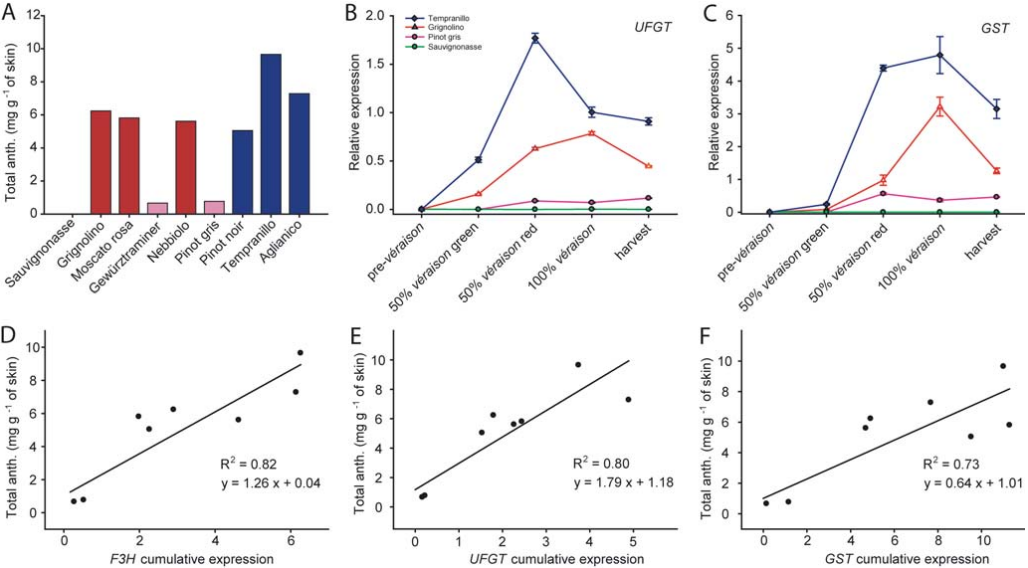
**Anthocyanin concentration and gene expression in berry skin**. (A) Total anthocyanins in nine cultivars. Concentration is expressed as mg g^-1 ^of skin of malvidin equivalents; (B) *UFGT *and (C) *GST *gene expression in four cultivars with no, low, medium and high amount of total anthocyanins at four ripening stages. At mid-*véraison *green berries were analysed separately from red berries on the same cluster; (D, E, F) linear regression between cumulative transcription of three genes (*F3H, UFGT *and *GST*) throughout ripening (calculated as the area below the expression curve) and final anthocyanin content.

The anthocyanin content in ripe berries of the pigmented cultivars ranged from 0.7 to 9.7 mg g^-1 ^of skin (Table 1, Figure 1A). Anthocyanins were not detected in berry skin of the white (green/yellow-skinned) cultivar 'Sauvignonasse', synonym for 'Tocai friulano'. Total anthocyanin content was less than 1 mg g^-1 ^of skin in the palely pigmented cultivars 'Gewürztraminer' and 'Pinot gris', ranged from 5.0 to 6.2 mg g^-1 ^of skin in 'Grignolino', 'Moscato rosa', 'Nebbiolo' and 'Pinot noir' and exceeded 7.3 mg g^-1 ^of skin in the dark pigmented cultivars 'Aglianico' and 'Tempranillo'. The cumulative transcription of *UFGT*, the specific gene for anthocyanin biosynthesis, calculated as the area below the curve of expression throughout ripening, increased proportionally to the anthocyanin content (Table 1) and showed a strong correlation (R^2 ^= 0.80) with the final anthocyanin content (Figure 1E). The kinetics of anthocyanin accumulation through four ripening stages showed overlapping patterns with the curve of expression of *UFGT*. Four examples of the expression profile of *UFGT *in a cultivar that does not synthesise anthocyanins ('Sauvignonasse'), a cultivar in which anthocyanins are barely detectable ('Pinot gris'), a cultivar that has an intermediate amount of anthocyanins ('Grignolino') and a cultivar rich in anthocyanins ('Tempranillo') are reported in Figure 1B. In all but one fully coloured cultivars ('Pinot noir', 'Tempranillo', 'Nebbiolo', Moscato rosa' and 'Grignolino'), expression profile of *UFGT *peaked between mid- and full-*véraison*. Transcript levels of the same genes were undetectable in 'Sauvignonasse' and barely detectable in the palely coloured cultivars 'Pinot gris' and 'Gewürztraminer'. The peak of *UFGT *gene expression was delayed in the cultivar 'Aglianico' compared to all other cultivars (data not shown). Sugar accumulation, burst of organic acids, expression of *DWF1*, a gene involved in the brassinosteroid-dependent promotion of ripening, and of other anthocyanin genes were also delayed in 'Aglianico'. The cultivation of this variety is more suited to warmer climates and it did not attain complete phenolic maturation at the site of this trial. All other cultivars displayed curves of anthocyanin accumulation and patterns of gene expression for *DWF1 *and *UFGT *as their requirements of growing degree days (GDD) had already been met at the moment of harvest.

**Table 1:** Anthocyanin content in full ripe berries and cumulative gene expression of anthocyanin genes throughout ripening in nine cultivars

	SA	GR	MR	GE	NE	PG	PN	TE	AG
Total anthocyanins (mg g^-1 ^skin)	-	6.2	5.8	0.7	5.6	0.8	5.0	9.7	7.3
% 3-G	-	76.0	78.4	38.5	84.1	71.4	92.4	73.6	77.6
% acetyl-3-G	-	16.5	13.4	50.6	8.9	26.0	7.5	8.2	6.4
% p-coumaryl-3-G	-	7.4	8.2	10.9	7.0	2.7	0.0	18.1	16.0
% PT-3-G	-	0.9	1.8	4.9	3.7	0.9	4.9	19.4	12.4
% C-3-G	-	12.5	8.2	67.0	9.1	2.1	1.6	2.6	1.0
% D-3-G	-	1.1	2.2	4.4	3.7	3.0	5.8	15.5	12.0
% PN-3-G	-	73.0	66.2	5.3	56.4	43.4	23.4	7.7	5.8
% M-3-G	-	12.6	21.5	18.4	27.1	50.7	64.3	54.8	68.8
% 3'4'-OH 3-G	-	85.5	74.4	72.3	65.5	45.4	25.0	10.3	6.8
% 3'4'5'-OH 3-G	-	14.5	25.6	27.7	34.5	54.6	75.0	89.7	93.2
% PN-3-G/3'4'-OH 3-G	-	85.4	89.0	7.4	86.1	95.5	93.6	74.8	85.3
% M-3-G/3'4'5'-OH 3-G	-	86.4	84.3	66.6	78.6	92.9	85.7	61.1	73.8
% PT-3-G/3'4'5'-OH 3-G	-	7.5	8.6	15.7	10.6	5.5	7.7	17.2	12.9
% (M-3-G + PT-3-G)/3'4'5'-OH 3-G	-	93.9	92.9	82.3	89.2	98.3	93.4	78.4	86.7
% (PN-3-G + PT-3-G + M-3-G)/3-G	-	86.5	89.6	28.7	87.3	95.0	92.6	81.9	87.0
Cumulative expression									
*F3H*	0.17	2.90	1.98	0.27	4.62	0.52	2.26	6.26	6.14
*UFGT*	-	1.79	2.44	0.16	2.26	0.22	1.53	3.74	4.89
*GST*	-	4.91	11.28	0.14	4.69	1.16	9.52	11.00	7.67
*F3'H*	0.01	0.07	0.12	0.02	0.04	0.02	0.03	0.10	0.12
*F3'5'H*	-	0.06	0.15	0.00	0.06	0.04	0.33	0.92	1.63
*OMT*	-	2.08	2.74	0.01	2.18	0.87	4.62	5.13	7.33
*MybA*	-	2.78	3.78	1.91	3.32	1.03	1.10	1.63	2.11
*MybB*	0.02	0.07	0.18	0.11	0.04	0.10	0.16	0.08	0.15
*MybC*	0.02	0.03	0.07	0.01	0.07	0.03	0.03	0.10	0.03
*MybD*	0.01	0.07	0.03	0.01	0.04	0.02	0.02	0.03	0.04

**Figure 2:**
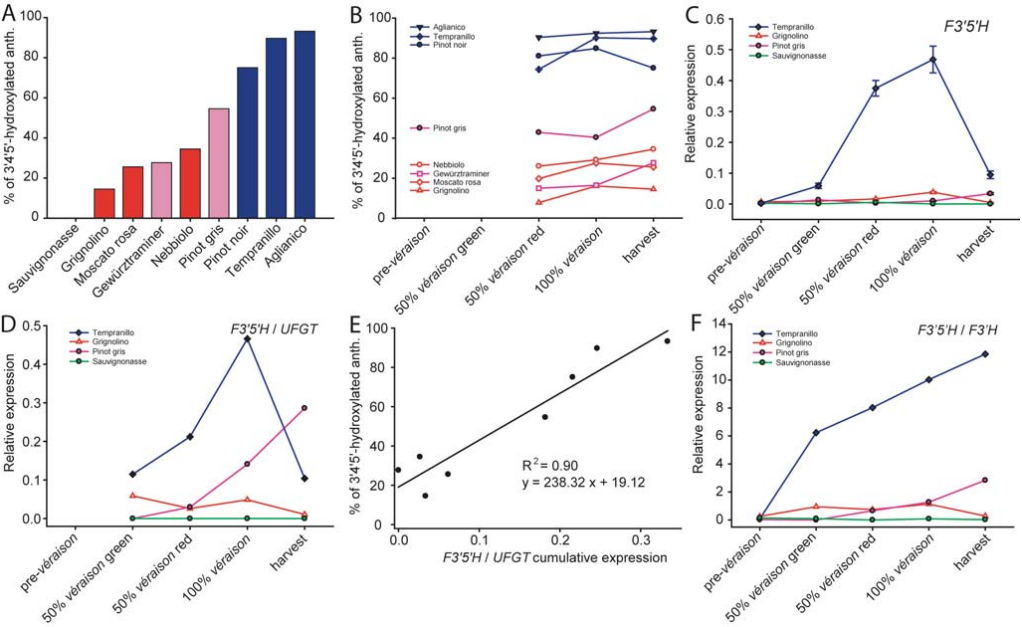
**Anthocyanin hydroxylation and expression of flavonoid 3',5'-hydroxylases**. (A) Percentage of tri-hydroxylated anthocyanins among 3-monoglucoside anthocyanins; (B) evolution of anthocyanin composition from the onset of coloration to full maturity; (C) *F3'5'H *gene expression in four cultivars with no, low, medium and high percentage of tri-hydroxylated anthocyanins at four ripening stages. At mid-*véraison *green berries were analysed separately from red berries on the same cluster; (D) pattern of *F3'5'H *gene expression normalised to the expression level of the anthocyanin biosynthetic gene *UFGT *(*F3'5'H */*UFGT*); (E) linear regression between the cumulative *F3'5'H */*UFGT *ratio and the final percentage of tri-hydroxylated anthocyanins; (F) pattern of *F3'5'H *gene expression normalised to the expression level of *F3'H *(*F3'5'H */*F3'H*).

Total anthocyanins, percentage of 3-monoglucoside (% 3-G), acetyl-3-glucoside (% acetyl-3-G) and *p*-coumaryl-3-glucoside (% *p*-coumaryl-3-G), percentage of single monoglucoside anthocyanins (petunidin, % PT-3-G; cyanidin, % C-3-G; delphinidin, % D-3-G; peonidin, % PN-3-G; malvidin, % M-3-G), percentage of di-hydroxylated monoglucoside anthocyanins (% 3'4'-OH 3-G) and tri-hydroxylated monoglucoside anthocyanins (% 3'4'5'-OH 3-G), percentage of peonidin among di-hydroxylated anthocyanins (% PN-3-G/3'4'-OH 3-G), percentage of malvidin and/or petunidin among tri-hydroxylated anthocyanins (% M-3-G/3'4'5'-OH 3-G), (% PT-3-G/3'4'5'-OH 3-G) and [(M-3-G + PT-3-G)/3'4'5'-OH 3-G], percentage of all methoxylated anthocyanins among 3-glucoside anthocyanins [(PN-3-G + PT-3-G + M-3-G)/3-G] and cumulative expression of six genes of the anthocyanin biosynthetic pathway (*F3H*, *F3'H*, *F3'5'H*, *UFGT*, *OMT*, *GST*), along with four transcription factors (*MybA*, *MybB*, *MybC*, *MybD*) in eight pigmented cultivars and one white cultivar ('Sauvignonasse'). Cumulative transcription of each gene from the onset of *véraison *to full maturity was calculated as the area below the expression curves as those reported in Figure 1B, 1C, 2C, 2D, 4B and 4C. SA, Sauvignonasse; GR, Grignolino; MR, Moscato rosa; GE, Gewürztraminer; NE, Nebbiolo; PG, Pinot gris; PN, Pinot noir; TE, Tempranillo; AG, Aglianico; -, not detectable.

The gene *GST *showed an expression pattern similar to that of *UFGT *(Figure 1C), even though the correlation between cumulative transcription throughout ripening and final amount of anthocyanins was slightly lower (R^2 ^= 0.73) (Figure 1F). The cumulative transcription of *F3H *was also strongly correlated with the final anthocyanin content (R^2 ^= 0.81) (Figure 1D). None of the four transcription factors *MybA*, *MybB*, *MybC*, and *MybD *showed strong correlation between cumulative transcription (Table 1) and total anthocyanin content at harvest (R^2 ^< 0.52).

### Anthocyanin profile at full maturity

The composition of 3-monoglucoside, acetyl-3-glucoside and *p*-coumaryl-3-glucoside anthocyanins in each cultivar is reported in Table 1. With regard to the acylation of the glycosyl group, non-acylated anthocyanins were the most abundant fraction in all cultivars except 'Gewürztraminer', in which acetyl-3-glucosides (50.6 %) predominated. The contribution of *p*-coumaryl forms was the lowest in all cultivars except 'Aglianico' and 'Tempranillo'. 'Pinot noir' was the only cultivar that synthesised exclusively monoglucoside (92.4 %) and acetyl-3-glucoside (7.6 %) anthocyanins, while *p*-coumaryl anthocyanins were not detected in the berry skin of this cultivar.

The contribution of each anthocyanin (cyanidin, peonidin, delphinidin, petunidin, malvidin) to the final anthocyanin profile was calculated based on the monoglucoside forms and is expressed as percentage in Table 1. Malvidin was the most abundant anthocyanin in all cultivars that had a prevalence of tri-hydroxylated anthocyanins. By contrast, among the cultivars that had a predominance of di-hydroxylated anthocyanins, peonidin was the most abundant in 'Grignolino', 'Moscato rosa' and 'Nebbiolo', while cyanidin was the most abundant in 'Gewürztraminer'.

### Hydroxylation of the B-ring and evolution of anthocyanin composition during ripening

Anthocyanin profiles differed dramatically among the nine cultivars studied. The abundance of blue tri-hydroxylated anthocyanins in ripe berries ranged from the lowest extreme of 14.5 % in 'Grignolino' to the highest extreme of 93.2 % in 'Aglianico' (Table 1 and Figure 2A). 'Tempranillo' and 'Pinot noir' also had a remarkable prevalence of tri-hydroxylated anthocyanins (89.7 and 70.0 %, respectively). Tri-hydroxylated derivatives roughly equalled di-hydroxylated derivatives in 'Pinot gris'. By contrast, 'Grignolino', 'Moscato rosa', 'Gewürztraminer' and 'Nebbiolo' had more di-hydroxylated than tri- hydroxylated anthocyanins. The evolution of the anthocyanin profile during ripening is reported in Figure 2B. Cyanidin-based pigments accumulated more promptly at the first sampling stage after the onset of colouration but their contribution to the total anthocyanin content declined as ripening proceeded, to the advantage of a more abundant synthesis of delphinidin-based anthocyanins. Whatever the final percentage in each cultivar, the contribution of tri-hydroxylated anthocyanins was higher at harvest than at the onset of *véraison *in all pigmented cultivars but 'Pinot noir'.

**Figure 3:**
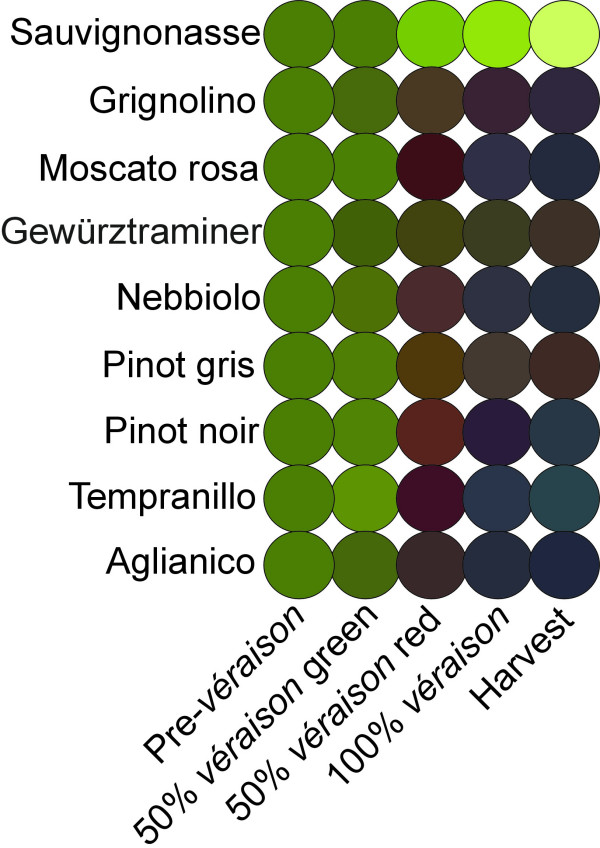
**Colour evolution of berry skin in nine cultivars**. Colour was reproduced by an image editing software (CorelDraw) using mean *L*, *a*, *b *values averaged on 70 berries at all ripening stages investigated in this study. At mid-*véraison *green berries were analysed separately from red berries on the same cluster.

The expression pattern of *F3'5'H *throughout ripening is shown in Figure 2C using four examples of a cultivar that does not synthesise anthocyanins ('Sauvignonasse'), a cultivar in which 3'4'-OH anthocyanins predominate ('Grignolino'), a cultivar in which 3'4'-OH and 3'4'5'-OH equally contribute to final anthocyanin content ('Pinot gris') and a cultivar very rich in 3'4'5'-OH anthocyanins ('Tempranillo'). We also normalised the expression of *F3'5'H *to the rate of anthocyanin biosynthesis by dividing the transcript level of *F3'5'H *by the transcript level of *UFGT *(*F3'5'H */*UFGT*). The evolution of the ratio *F3'5'H */*UFGT *through ripening is reported in Figure 2D. The cumulative ratio *F3'5'H */*UFGT *calculated from the onset of ripening to harvest was strongly correlated with the final percentage of tri-hydroxylated anthocyanins (R^2 ^= 0.90) (Figure 2E). We also calculated the expression ratio between *F3'5'H *and *F3'H*, shown here in four representative cultivars (Figure 2F). Colour of berry skin in cultivars in which *F3'5'H *transcription was highly activated showed a shift to blue, in agreement with higher relative abundance of tri-hydroxylated anthocyanins. Skin colour evolution is represented in Figure 3. Colour was reproduced by an image editing software (CorelDraw) using mean *L*, *a*, *b *values averaged on 70 berries at all ripening stages investigated in this study.

### Methoxylation of the B-ring

**Figure 4:**
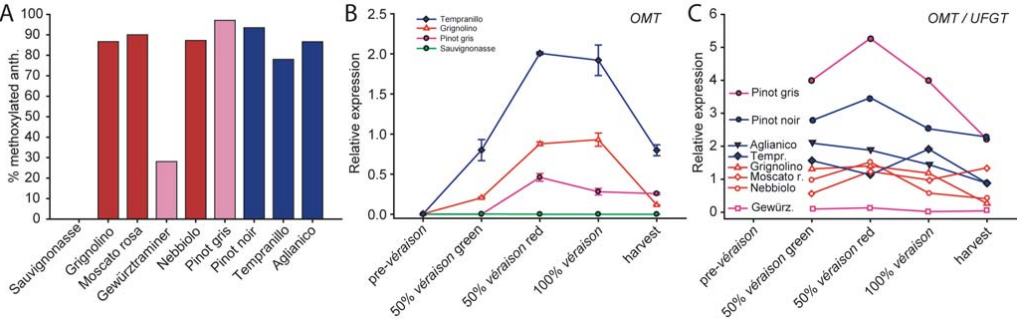
**Anthocyanin methoxylation and expression of *O*-methyltransferase**. (A) Percentage of methoxylated anthocyanins (peonidin, petunidin and malvidin) among 3-monoglucoside anthocyanins; (B) *OMT *gene expression in four reference cultivars at four ripening stages. At mid-*véraison *green berries were analysed separately from red berries on the same cluster; (C) pattern of *OMT *gene expression normalised to the expression level of the anthocyanin biosynthetic gene *UFGT *(*OMT */*UFGT*) in all cultivars.

The contribution of methoxylated (peonidin, petunidin and malvidin) and non-methoxylated (cyanidin and delphinidin) anthocyanins to the final profile was calculated in the genotypes studied (Figure 4A). 'Gewürztraminer' had the lowest percentage of methoxylated anthocyanins (28.1 %) whilst all other pigmented cultivars had a percentage of methoxylated forms higher than 78 %. In particular, 'Pinot gris' and 'Pinot noir' scored the highest percentages of 97.1 and 93.4 %, respectively. 'Grignolino', 'Moscato Rosa', 'Nebbiolo' and 'Aglianico' had percentages of methoxylated anthocyanins ranging between 86 and 90 %; in 'Tempranillo' the percentage was lower than 80 %. The percentage of each methoxylated derivative calculated among the corresponding hydroxylated form is reported in Table 1. The expression pattern of *OMT *in four reference cultivars, 'Sauvignonasse', 'Pinot gris', 'Grignolino' and 'Tempranillo', is reported in Figure 4B. The relative expression of *OMT *was normalised to the rate of anthocyanin biosynthesis by dividing the transcript level of *OMT *by the transcript level of *UFGT *(*OMT */*UFGT*). The evolution of the ratio of transcriptional level *OMT */*UFGT *through ripening and the relative abundance of methoxylated anthocyanin is compatible with a role of OMT in the methoxylation of the B-ring (Figure 4C).

## Discussion

Anthocyanin pigmented grapes appear in many stunning colour variations. In spite of the invariable presence of all anthocyanin biosynthetic genes in any investigated cultivar, a genotype-specific regulation of the genes along the core pathway and at the main branching points is presumed to underlie the observed quantitative variation in anthocyanin content and the red-to-blue shift in anthocyanin pigmentation [26]. In the experiment presented in this paper, differences in anthocyanin pigmentation across fruit of nine grapevine cultivars are attributable to variations of the peculiar pattern of expression for the global set of anthocyanin genes. Gene expression of *F3H*, *UFGT*, *OMT*, *GST *strongly increased at *véraison *in all cultivars except for the non-pigmented 'Sauvignonasse', and the palely pigmented 'Gewürztraminer' and 'Pinot gris'. In the latter two palely pigmented cultivars, the levels of transcripts remained low although detectable throughout ripening. Striking differences were also observed in the regulation of flavonoid hydroxylases. Expression profile of *F3'H *was relatively high even before the onset of anthocyanin biosynthesis, and transcripts of this gene were present after *véraison *in all cultivars, also including the white cultivar 'Sauvignonasse'. By contrast, transcription of *F3'5'H *was developmentally activated after the onset of *véraison *in 'Aglianico', 'Tempranillo' and 'Pinot noir', the cultivars that synthesise mostly 3'4'5'-hydroxylated anthocyanins and bear blue-skinned berries, while it remained at lower levels in the red cultivars and was not transcribed at all in 'Sauvignonasse'. Cumulative mRNAs of *F3H *and *UFGT *throughout ripening and relative abundance of *F3'5'H *to *UFGT *and of *OMT *to *UFGT *explained *per se *large part of the phenotypic variation for anthocyanin content, anthocyanin composition, colour intensity and colour hue of grapes at berry maturity. Most regulation of the flavonoid pathway has been shown to occur at transcriptional level also in pigmented organs other than fruit in other species [1,6,27,28]. In *Z. mays *kernels, the entire flavonoid pathway from chalcone synthase downwards is simultaneously regulated by *Myb *type and basic helix-loop-helix transcriptional factors; in *A. majus *floral organs, the anthocyanin pathway is regulated as a block from the gene *F3H *downwards; in *P*. *hybrida *flowers, the pathway is regulated as a unit from the gene *DFR *downwards. In grapevines, the main control point for anthocyanin quantitative variation is downstream in the pathway at the UFGT level in agreement with the early observations by [29], but the variation in anthocyanin composition is finely tuned upstream of UFGT at the level of flavonoid hydroxylases (F3'H and F3'5'H) and downstream of UFGT at the level of *O*-methyltransferase. This would reflect a higher specialisation in partitioning flavonoid intermediates towards different classes of end-products (flavonols, catechins, various anthocyanins) which accumulate in the same tissue during different stages of berry development.

We have shown that colour variation in the grape fruit is directly dependent on changes in mRNA levels of the global set of anthocyanin enzymatic genes. Also in other species, mostly ornamentals, shifts in flower colour across genotypes are more frequently associated with changes in regulation of gene expression rather than with structural mutations that result in altered protein activity [30,31]. Variation in the expression of enzymatic genes might ultimately depend on regulatory genes that control transcription of some or all structural genes or on variation of *cis*-acting elements of the target structural genes that respond to the regulators [32]. We analysed gene expression of four *Myb*-type transcription factors known in grape, including the *MybA *gene that controls the transcriptional activation of *UFGT *[8]. The cumulative expression of none of the four transcription factors was sufficient *per se *to explain the quantitative variation in anthocyanin content, which probably conceals the presence of additional factors involved in the process. The synthesis of different classes of flavonoid compounds from common precursors occurs in the same cells within the grape skin. Hence, the few anthocyanin transcription factors investigated so far in grape may represent only the tip of the iceberg of a more complex regulatory network of the flavonoid pathway. Like in other species, it is possible that other *Myb*-type and basic helix-loop-helix transcriptional factors and WD40 proteins might differentially modulate the expression of structural genes of the early and late steps, of the core backbone and of side branches of the pathway, at different developmental stages and in different genotypes.

## Conclusion

Natural phenotypic differences offered us the opportunity to follow up the role of anthocyanin genes that lead to extreme colorations in grapes. We traced the determination of berry colour from the phenotypic level down to the transcriptional level through the metabolite level. The regulation of the anthocyanin pathway was peculiar to each cultivar. By following the expression profile from the onset of *véraison *till full maturity, it was possible to associate anthocyanin metabolites and discrete colour phenotypes with transcriptional profiles of structural genes. Further investigations are required to identify the suite of changes in regulatory elements across cultivars. Thanks to the privilege of being the first fruit crop to have its genome sequenced [33] and owing to the economical impact of grape anthocyanins for enological worth (production of premium red wines), nutraceutical value (dietary polyphenols) and dye industry uses (natural colorants), grapevines may become the archetype for studying anthocyanin regulation in fruit just as petunias have been for studying anthocyanin regulation in flowers.

## Methods

### Plant material

Vines were grown at the germplasm repository of Vivai Cooperativi Rauscedo, northeastern Italy (46° 04' N; 12° 50' E; 110 masl). Vines were trained to Sylvoz. Sampling was scheduled at four ripening stages: pre-*véraison *(on average at 4.2 °Brix of soluble solids and 37.1 g L^-1 ^titratable acidity), mid-*véraison *(50 % of coloured berries in pigmented cutlivars or 50 % of softened berries in 'Sauvignonasse' and 'Gewürztraminer'), 100 % *véraison*, full maturity (on average at 17.4 °Brix of soluble solids and 7.4 g L^-1^titratable acidity). Due to different thermal requirements for reaching the same phenological stage in different cultivars, complete *véraison *occurred in a window of time from July 28^th^, 2005 in 'Moscato rosa' to September 2^nd ^in 'Aglianico', 'Grignolino' and 'Nebbiolo' and technological maturity was reached from September 5^th ^in 'Pinot Noir', 'Moscato rosa', 'Pinot gris', and 'Gewürztraminer' till October 4^th ^in 'Aglianico' and 'Nebbiolo' [see Additional file 1]. At each stage, samples of 70 berries were collected for berry weight determination and colorimetric measurements. At mid-*véraison*, green berries were sampled separately from red berries on the same clusters. Berry colour was measured with an X-Rite 948 Chromameter (X-Rite). Colorimetric specification was referenced to the CIELab scale. Then, skin was peeled for anthocyanin extraction (see below) and pulp was used for quantifying total soluble solids and titratable acidity. Soluble solids were measured by a refractometer and expressed as °Brix, titratable acidity was expressed as tartaric acid equivalents. Forty more berries were sampled on the same clusters and peeled skins were used for RNA extraction.

### Anthocyanin quantification and profiles

Anthocyanins were extracted for 4 hours from 200 mg of berry skin with 2 mL of methanol, then centrifuged and filtered with a 0.2 μm PTFE filter (Chemtek analitica). After methanol evaporation, anthocyanins were re-suspended with 100–400 μL of 27:73 methanol:perchloric acid 0.3% (v/v). Anthocyanins were separated by HPLC using a C18 Purospher RP-18 (5μm, 250 mm × 4 mm) column (Merck), according to the procedure reported by [11]. Anthocyanin content was expressed as mg L^-1 ^of malvidin 3-glucoside. The composition of monoglucoside anthocyanins was used for calculating the percentage of 3'4'-OH and 3'4'5'-OH derivatives and the percentage of methoxylated anthocyanins.

### Transcript profiling

RNA extraction, DNase treatment and cDNA synthesis were performed as described in [11]. Quantitative real-time PCR was carried out on a DNA Engine Opticon2 (MJ Research) using SYBR Green. Each reaction (20 μL) contained 200 nM each primer, 1:60 (or 1:240) diluted cDNA, 0.4 U of HotMaster *Taq *polymerase (Eppendorf), 4.0 mM Magnesium Acetate, 0.4 mM dNTPs and SYBR solution (Eppendorf). Thermal cycling conditions were 95°C for 3 min followed by 94°C for 15 s, 56°C for 20 s, and 68°C for 30 s for 40 cycles, followed by a melting cycle from 65°C to 95°C. Each cDNA sample was analysed at two different dilutions (1:60 and 1:240 of the original cDNA), each dilution run in duplicate. Gene transcripts were quantified upon normalisation to Ubiquitin conjugating factor by comparing the cycle threshold (C_T_) of the target gene with that of *UbiCF *(CF203457.1, primer forward 5'-CTATATGCTCGCTGCTGACG, primer reverse 5'-AAGCCAGGCAGAGACAACTC). Gene expression was calculated as overall mean and standard error among all dilutions and replicates. Primers pairs for *UFGT *were retrieved from [34], *DWF1 *from [35], *GST *from [36], *F3H, F3'H, F3'5'H*, *OMT*, *MybA, MybB, MybC *and *MybD *from [11].

## Authors' contributions

SDC and GDG conceived the experiments and interpreted the results with equal contribution. SDC conducted field experiments, metabolite analysis and integrated metabolite and transcriptional data. GDG carried out expression analysis and drafted the manuscript. Both authors have read and approved the final manuscript.

## Supplementary Material

Additional file 1 **Phenology of the nine cultivars used in this study over the ripening period**. The data provided represent parameters of berry growth and juice composition at four stages of berry maturation. Click here for file
