# Supplementary material for: Transcriptional control of anthocyanin biosynthetic genes in extreme phenotypes for berry pigmentation of naturally occurring grapevines
Source: BMC Plant Biol. 2007 Aug 30;7:46. doi: 10.1186/1471-2229-7-46 (PMC2147006; doi:10.1186/1471-2229-7-46)
Supplement: Additional file 1 — Phenology of the nine cultivars used in this study over the ripening period. The data provided represent parameters of berry growth and juice composition at four stages of berry maturation. [file 1471-2229-7-46-S1.pdf]

Additional file 1 - Phenology of the nine cultivars used in this study over the ripening period

Cultivar	Pre-*vèraison*					50% *vèraison*					100% *vèraison*					harvest				
	Weight	°Brix	pH	Acidity	Date	Weight	°Brix	pH	Acidity	Date	Weight	°Brix	pH	Acidity	Date	Weight	°Brix	pH	Acidity	Date
Moscato rosa	0.92	7.2	2.29	33.5	26-Jul	1.99	8.2	2.35	29.5	29-Jul	2.74	13.6	2.90	11.8	12-Aug	2.76	19.2	3.23	7.5	05-Sep
Pinot gris	0.85	4.8	2.35	39.7	26-Jul	1.11	10.4	2.72	24.9	01-Aug	1.35	16.6	3.10	9.2	18-Aug	1.57	17.4	3.27	7.9	05-Sep
Pinot Noir	0.89	3.8	2.33	41.1	26-Jul	1.02	7.4	2.65	27.2	01-Aug	1.35	13.2	2.98	11.8	12-Aug	1.67	18.2	3.40	6.0	05-Sep
Gewurztraminer	1.32	3.8	2.55	36.7	26-Jul	1.19	5.2	2.80	26.8	01-Aug	0.77	12.8	3.12	11.6	18-Aug	1.91	16.4	3.46	5.5	05-Sep
Tempranillo	1.15	4.2	2.35	40.4	26-Jul	1.40	9.0	2.73	26.8	01-Aug	1.87	12.2	2.94	12.9	12-Aug	2.50	17.2	3.35	6.8	15-Sep
Sauvignonasse	0.66	3.4	2.33	28.9	26-Jul	1.02	6.2	2.57	32.1	08-Aug	1.27	11.2	2.83	14.7	17-Aug	1.31	16.8	3.37	7.1	15-Sep
Grignolino	0.93	3.0	2.38	32.5	26-Jul	1.18	8.6	2.66	25.8	12-Aug	1.68	16.0	3.01	10.8	02-Sep	1.72	17.4	3.25	7.6	15-Sep
Aglianico	0.87	3.2	2.34	34.9	26-Jul	1.82	8.0	2.49	35.3	18-Aug	2.32	13.0	2.72	18.1	02-Sep	2.38	15.9	2.98	9.5	04-Oct
Nebbiolo	1.26	3.4	2.26	38.3	26-Jul	1.62	9.8	2.62	29.4	18-Aug	2.11	13.6	2.85	15.1	02-Sep	2.35	18.0	3.12	8.7	04-Oct
